# Near‐fatal bleeding due to ruptured peripheral varicose vein that developed after previous orthopedic surgery: A case report

**DOI:** 10.1002/ccr3.8139

**Published:** 2023-11-12

**Authors:** Jumpei Takamatsu, Hajime Nakajima, Masatoshi Nakata, Aya Fukuhara, Jinkoo Kang, Yuichi Yasue

**Affiliations:** ^1^ Department of Emergency Medicine and Critical Care Kansai Rosai Hospital Amagasaki Hyogo Japan

**Keywords:** hemorrhage, lower extremities, organizing thrombus, surgery, varicose veins

## Abstract

This case highlights the necessity to inform patients with vein ligation about the possibility of varicose vein formation in the periphery and brings awareness to emergency staff that bleeding could be caused by a ruptured peripheral varicose vein.

## Graphical abstract

The structure of the vein wall disrupted by a structure (▲), which appears to be a thread, and the rupture of the elastic fibers (△).

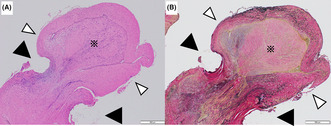

## 1 INTRODUCTION

Lower extremity peripheral venous relaxation results from a malfunction of the venous valves in the saphenous veins, leading to regurgitation, stagnation, or retention of venous blood in the saphenous vein branches. This results in swollen, visible peripheral varicose veins on the skin surface, which are associated with swelling, pain, severe skin discoloration, and potential ulceration.^1^ Peripheral varicose veins are often asymptomatic and, if not sufficiently large enough, are likely to be unnoticed. However, with the rising aging population, an increased number of bleeding cases due to age‐related vascular fragility have been reported.^2^, ^3^ The number of people who easily fracture their lower limbs due to falls and other causes and those receiving anticoagulants is also increasing. Moreover, even small varicose veins can lead to massive bleeding and fatal outcomes. One case reported that a 91‐year‐old woman, who was self‐sufficient and living alone, took oral antiplatelet drugs, and died of ruptured varicose veins at home.^4^ Herein, we present a case of severe hemorrhage from rupturing of peripheral varicose vein that were formed due to previous surgery. Therefore, emergency physicians should consider patients with this disease when they encounter hemorrhage shock due to bleeding from a non‐traumatic extremity. Moreover, family physicians should consider discussing the long‐term risks of massive bleeding from the procedure with the patient with previous extremity surgery.

## 2 CASE PRESENTATION

**FIGURE 1:**
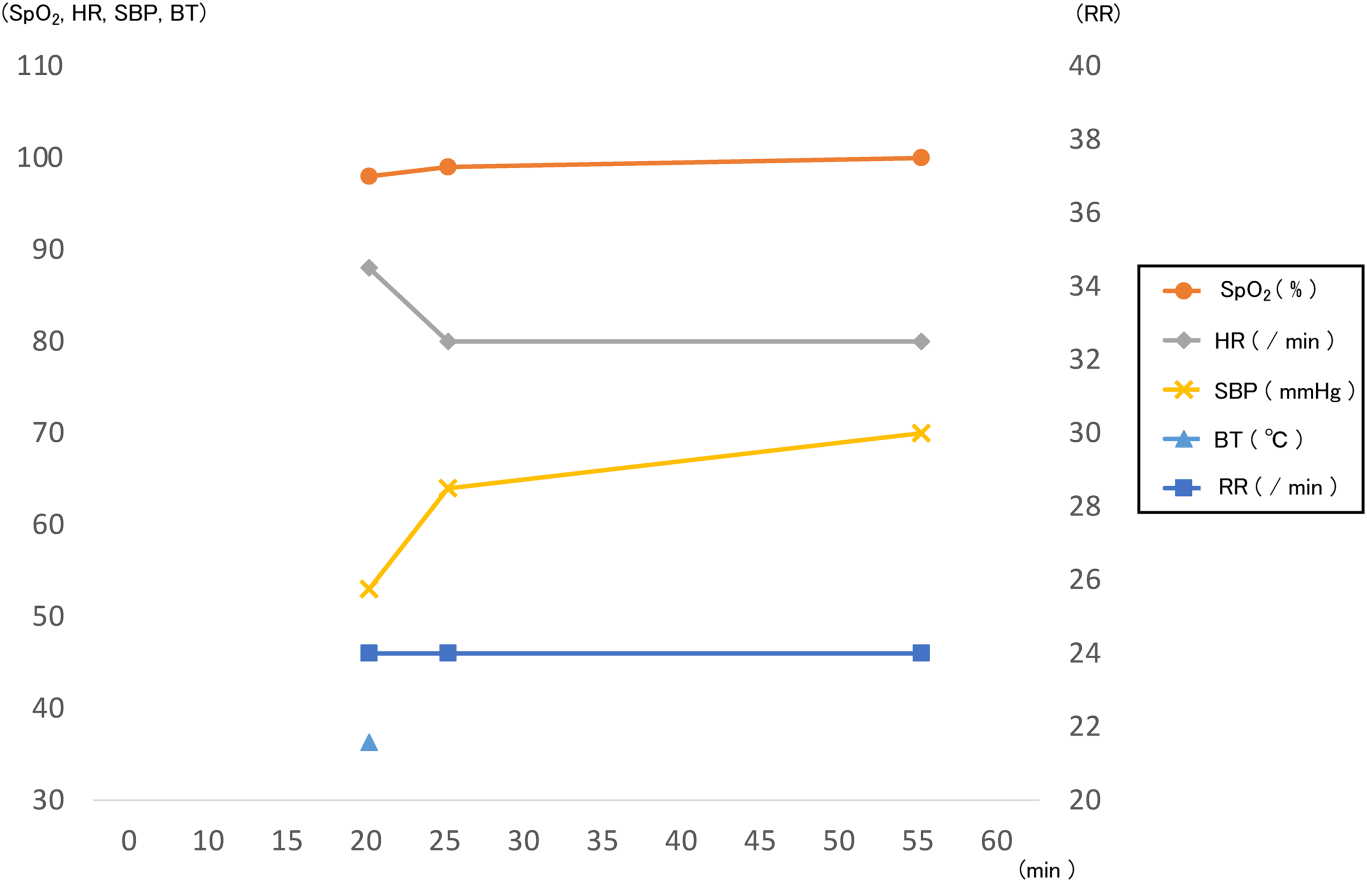
Clinical course results in the ambulance.

An 86‐year‐old male patient suddenly began bleeding profusely from the left ankle joint while working in his restaurant. The patient was taking carvedilol (10 mg/day) for hypertension and had undergone open repair and fixation of a distal end fracture of the left tibial fibula at another hospital 10 years prior. However, he had no past medical or family history of bleeding disorders. On checking vital signs, the systolic blood pressure had dropped to 50 mmHg, and the patient was in shock. Figure 1 depicts the sequence of events from the time of contact with the patient until he arrived at the hospital. A doctor's car was requested and docked on the way to transport the patient. His level of consciousness (Glasgow Coma Scale) was E (eye‐opening) 3, V (best verbal response) 4, M (best Motor response) 5; the respiratory rate was 20 breaths/min, and SpO_2_ was 99% (oxygen mask with reservoir, 10 L/min). He had pallor, marked coldness, marked sweating, blood pressure of 64/55 mmHg, and pulse rate of 83 beats/min. Arterial blood gas analysis revealed a lactate level of 5.9 mmol/L. At the scene, active bleeding was not noted, and the cause could not be identified in the ambulance because of adherent blood. After docking, the peripheral venous tract was secured, fluid was administered, and a tourniquet was wrapped around the left lower leg to prevent further bleeding.

**FIGURE 2:**
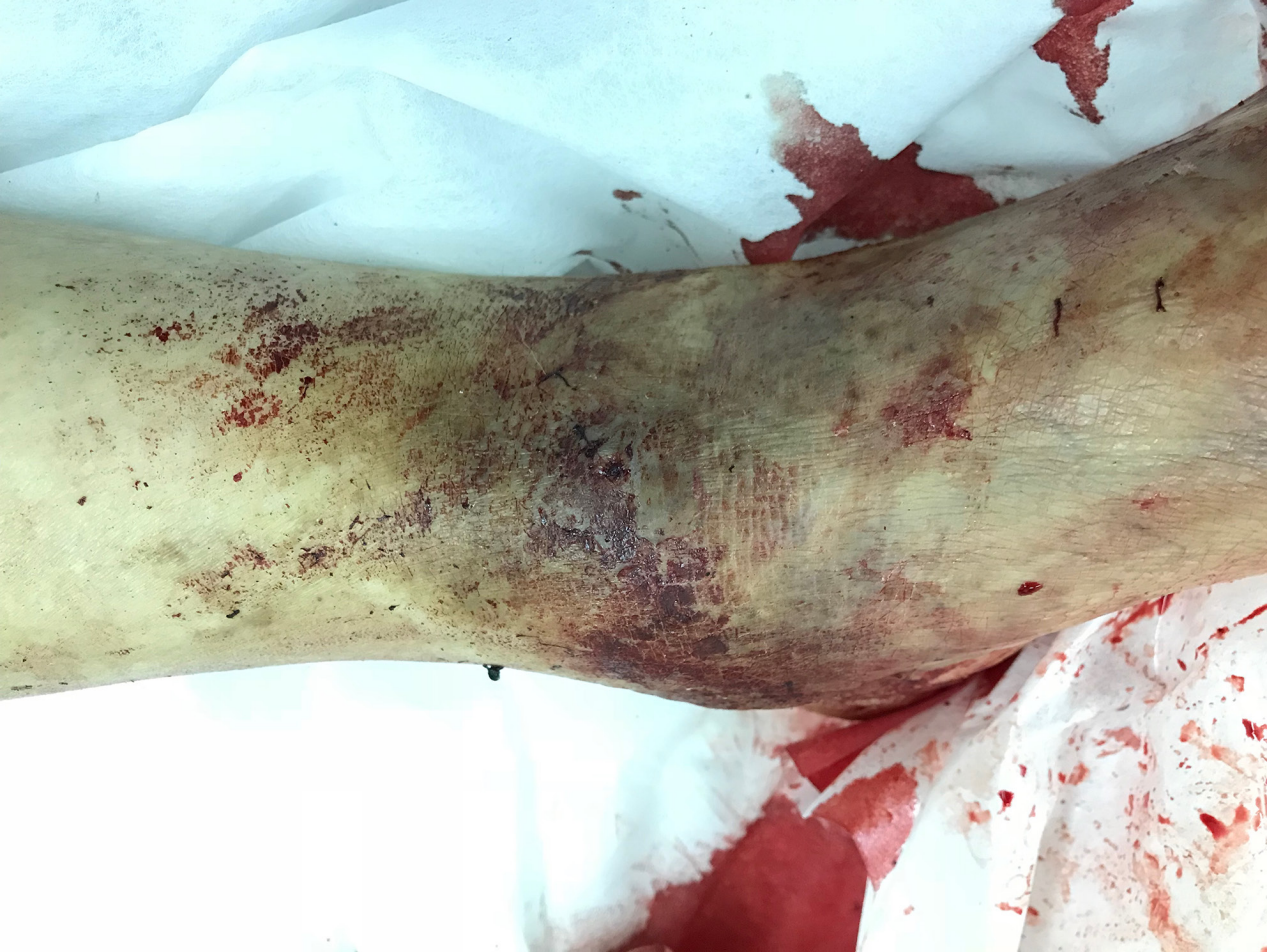
Removal of adherent blood revealed an ulcer, considered the source of bleeding.

**FIGURE 3:**
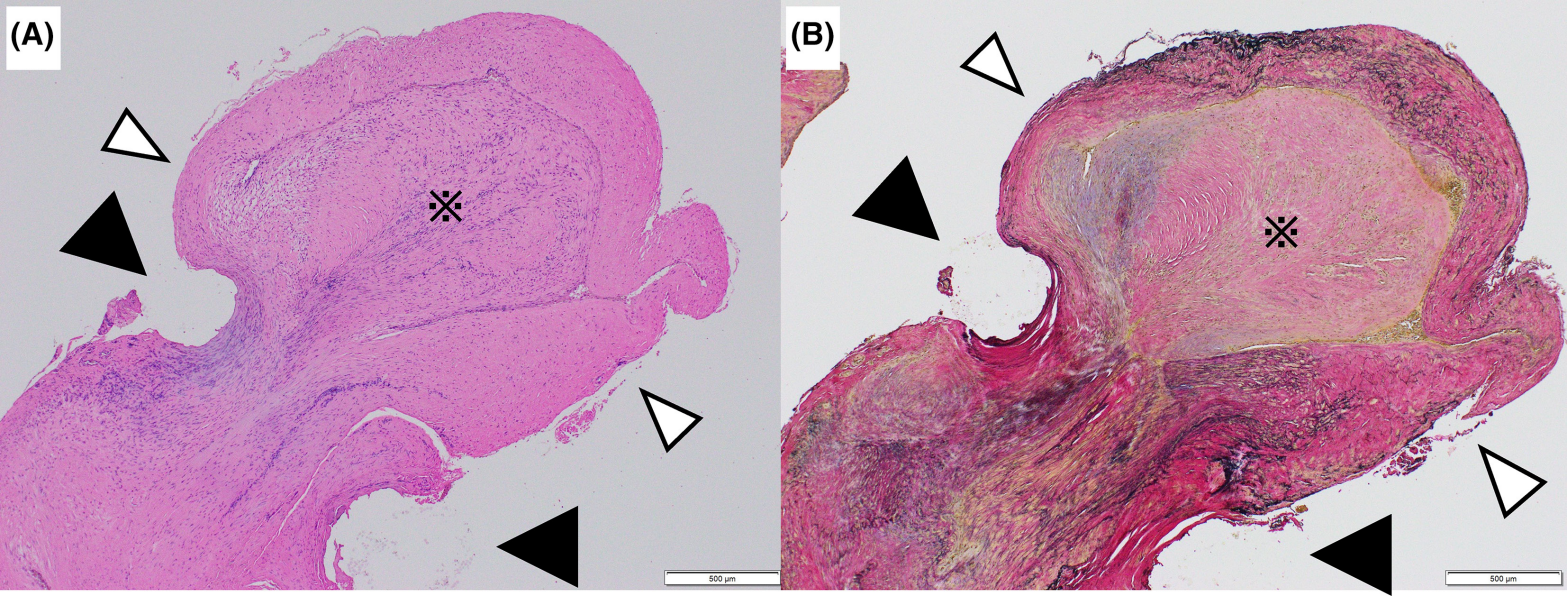
Histological findings. (A) Hematoxylin–eosin staining. (B) Elastica van Gieson staining. They show that the structure of the vein wall is disrupted by a structure (▲), which appears to be a thread and a rupture of the elastic fibers (△). As a result, an organized thrombus (※) is formed in the vessel lumen.

**FIGURE 4:**
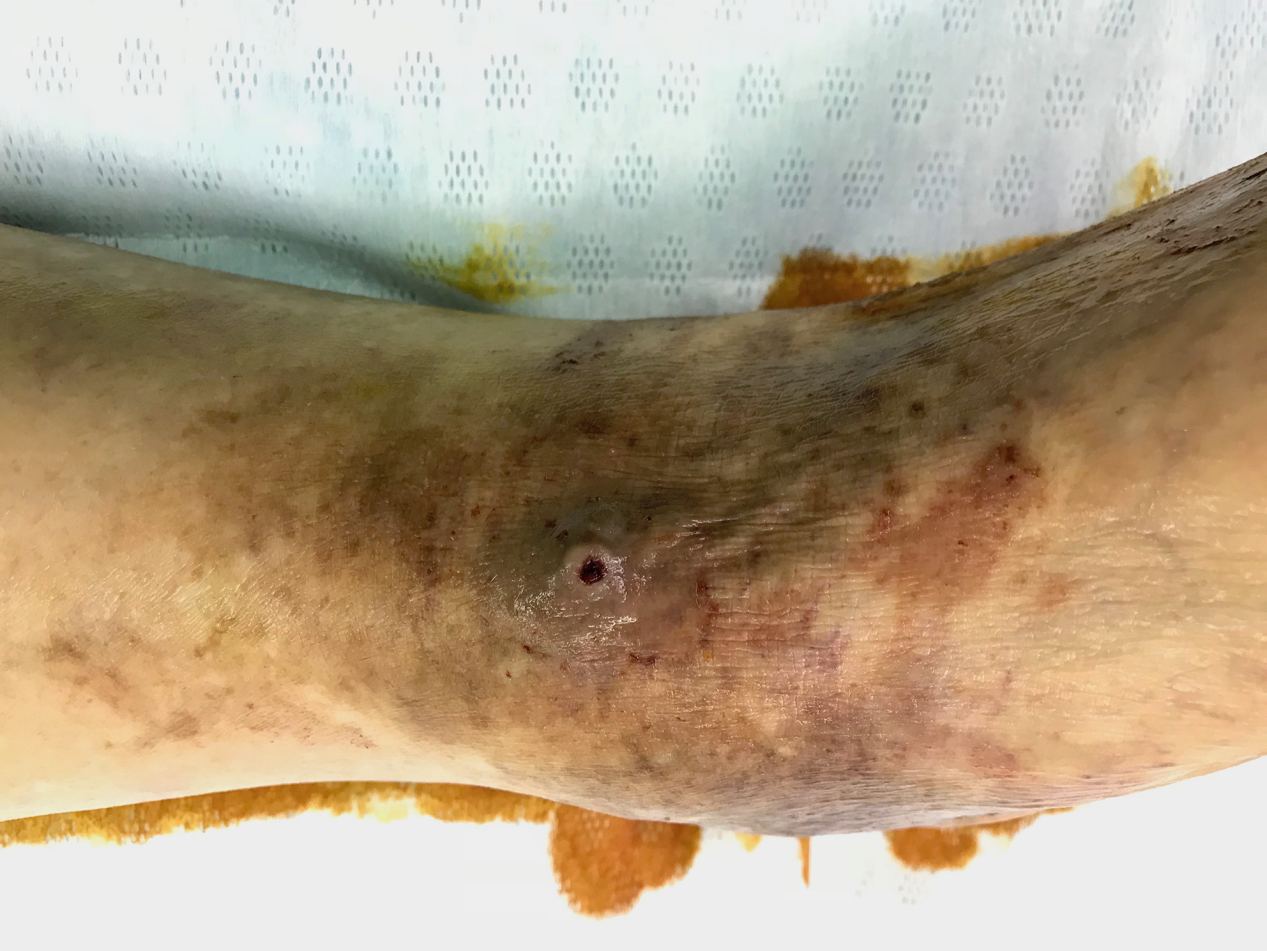
The ulcer healed, and the varicose vein disappeared.

Upon hospital admission, the patient was somnolent; therefore, we administered crystalloid fluid (500 mL and plasma protein). His blood pressure gradually stabilized, and his consciousness improved. After cleaning the blood from his left lower leg, a 1‐cm varicose vein was found on the left dorsal surface with a 5‐mm ulcer at its apex (Figure 2). On his laboratory data, anemia was not yet apparent. Lactic acid was elevated. No coagulation abnormalities were noted (Table 1). To prevent rebleeding, emergency high‐level ligation of the varicose vein was performed, and the ruptured varicose vein was resected, including the ulcer area. The histopathological examination indicated that the vein wall of the excised specimen was markedly thickened with a dilated lumen, which was consistent with varicose vein (Figure 3). However, a portion of the vein was occluded by an organizing thrombus due to a tear in the tunica media caused by a suture from the previous surgery. The postoperative course was uneventful; the ulcer closed, and the patient was discharged on the second postoperative day (Figure 4). At 3 months postoperatively, there had been no recurrence.

**TABLE 1:** Laboratory data.

Item	Result
White blood cells (×10^3^/μL)	7.0
Red blood cells (×10^6^/μL)	3.42
Hematocrit (%)	31.5
Platelet (×10^3^/μL)	197
International normalized ratio of prothrombin time	1.11
d‐dimer (μg/mL)	0.73
Total bilirubin (mg/dL)	0.4
Aspartate aminotransferase (U/L)	14
Alanine aminotransferase (U/L)	10
Lactate dehydrogenase (U/L)	164
Sodium (mmol/L)	140
Chloride (mmol/L)	103
Potassium (mmol/L)	3.5
Blood urea nitrogen (mg/dL)	25.4
Creatinine (mg/dL)	1.23
Albumin (U/L)	3
Lactate (mmol/L)	5.7
C‐reactive protein (mg/dL)	0.2
PCT (ng/mL)	0.07

## 3 DISCUSSION

Peripheral varicose veins are a chronic condition. However, a varicose vein of an imperceptible size can rupture and cause fatal bleeding. The legs are more prone to varicose veins, and a previous surgery can cause varicose veins to form and worsen in the same area. Therefore, patients should be informed about the possibility of varicose vein formation in the periphery of the surgical area if vein ligation is performed. Two types of ruptured varicose veins can cause fatal bleeding: the acute perforation type, in which the ulcer is less than 5 mm and is not accompanied by skin lesions, and the other type, which has the chronic ulcerative ulcers (more extensive and profound than the previous ones).^5^ In the present case, the size of the bleeding spot was very small (5 mm). Although pigmentation was observed around the spot, it was considered an acute perforation type with no other lesions. The characteristics of deaths due to massive hemorrhage from ruptured varicose veins include aging, social status, severe sequelae or dementia, hemorrhage related to minor trauma, rapid disease course, alcohol consumption, and anticoagulant use.^3^ Another important risk factor is sclerotic changes in vessel walls, which can lead to spontaneous bleeding.^2^ In addition, complications, such as ischemic heart disease^6^ and cirrhosis, chronically impair the liver from producing coagulation factors and lead to significant bleeding and accelerated death.^7^ In other words, factors that exacerbate venous return, fragile vessels, or bleeding conditions can cause a fatal variceal rupture. In the present case, the only risk factor was aging; however, aging is a major cause of varicose vein formation.

The vulnerable leg veins in older people, who are more prone to varicose veins, can be exacerbated by previous surgical venous ligatures of the leg, which can lead to varicose vein formation at the same site. To date, no reports on the histopathologic features of ulcers suggest fatal variceal rupture due to previous surgical vein ligation.^8^ The pathological findings, in this case, demonstrated that venous ligation during fracture surgery resulted in impaired venous return and vein damage, which caused the varicose vein to rupture.

Therefore, patients should be informed that they might develop varicose veins in the periphery if venous ligation was performed in a previous surgery. The lack of adequate information and training related to this condition is the main reason for first aid being not performed by the patients themselves. Whenever a therapist treats a patient with varicose veins for other pathologies that may aggravate varicose veins, the possibility of harmful interactions with the pathological entity leading to fatal bleeding of the varicose veins must be considered. Additionally, patients with these risk factors must be educated regularly; the same is true for the present case.

## 4 CONCLUSION

In conclusion, we experienced a case of asymptomatic peripheral varicose vein rupture in a lower extremity that resulted in hemorrhagic shock due to sudden massive bleeding. This was a very rare case in which histopathology confirmed that previous surgery resulted in varicose veins formation. Thus, to prevent rupture, it is important to inform patients about potential fatality from a rupture. Moreover, orthopedic surgeons must be aware of this risk factor, and emergency physicians must consider this as a possible cause when handling bleeding cases.

## AUTHOR CONTRIBUTIONS

**Jumpei Takamatsu:** Conceptualization; data curation; project administration; writing – original draft. **Hajime Nakajima:** Conceptualization. **Masatoshi Nakata:** Conceptualization. **Aya Fukuhara:** Conceptualization; data curation. **Jinkoo Kang:** Conceptualization. **Yuichi Yasue:** Conceptualization.

## FUNDING INFORMATION

This study did not receive any grants from funding agencies in the public, commercial, or not‐for‐profit sectors.

## CONFLICT OF INTEREST STATEMENT

The authors declare that they have no competing interests.

## ETHICS STATEMENT

Ethical approval was waived by the institution.

## CONSENT

Written informed consent was obtained from the patient to publish this report in accordance with the journal's patient consent policy.

## Data Availability

The datasets used and/or analyzed during the current study are available from the corresponding author upon reasonable request.
